# Smog Avoidance Investment While Improving Air Quality: Health Demand or Risk Aversion? Evidence from Cities in China

**DOI:** 10.3390/ijerph18157788

**Published:** 2021-07-22

**Authors:** Jichun Zhao, Hongbiao Wang, Jianxin Guo

**Affiliations:** Institute of Agricultural Information and Economics, Beijing Academy of Agricultural and Forestry Sciences, Beijing 100097, China; zhaojc@agri.ac.cn (J.Z.); wanghb@agri.ac.cn (H.W.)

**Keywords:** smog avoidance behavior, health care expenditure, health production efficiency hypothesis, time preference hypothesis

## Abstract

Atmospheric pollution control policies have achieved remarkable progress in China since 2013, and the smog protective equipment market has experienced a great boom during the same period. From the perspectives of the health production efficiency hypothesis and the time discount rate hypothesis, this study investigates the relationship between household expenditures on air pollution avoidance and health care, and individuals’ self-assessed health based on network survey data from 17 cities in China. Using the treatment effect model to control the potential endogenous selection problems, we explain the paradox of the growing smog avoidance investment coexisting with improving air quality. First, smog avoidance investment and household medical expenditures do not have substitution effects, while the perception of pollution intensity, pollution protection knowledge, and future health preferences significantly promote smog avoidance investment and medical expenditures. Second, air pollution avoidance investment greatly increases the probability that urban residents rate their health as “good” and “very good”. The results indicate that the time preference hypothesis can explain the pollution avoidance investment behavior and health demands of Chinese urban residents well. The hidden social welfare loss caused by air pollution may still be underestimated, even though short-term avoidance costs are included in the evaluation of pollution impacts. It is necessary to optimize environmental regulations and policies to consistently improve the ecological environment.

## 1. Introduction

On 10 September 2013, the State Council issued the “Air Pollution Prevention and Control Action Plan” (shorter form, the “Ten Rules of Qi”), which implemented comprehensive prevention and control measures, such as eliminating backward production facilities, setting emission limits for high-polluting industries, and implementing contract energy management for 47 cities in “three regions and 10 clusters”. With the central government’s subsequent promotion of the supervision system for environmental protection and air quality, air pollution control in relevant areas was strengthened to an unprecedented extent, and the “blue sky defense war” achieved remarkable progress. Surprisingly, a paradox has emerged in households’ health capital investment, that is, households’ investment in smog avoidance is growing with the improvement of air quality. The smog avoidance investment mainly concerns the purchase of anti-smog masks and air purifiers. Anti-smog masks are used to reduce outdoor exposure, and air purifiers are aimed at reducing the concentration of indoor air pollutants. The sales of masks in China exceeded 5 billion pieces (27 billion RMB) in 2019 and the sales amount of air purifiers increased to 20 billion RMB in 2017, exceeding 7 million units, with an average compound growth rate of approximately 40%. From the perspective of the smog protection product market price in 2017, the expenditure of using masks or air purifiers alone is 7.9–33.8% of the household health care expenditure for a standard family (three-person family) in China, which has actually become an important expenditure. The aim of this paper is to explore the causes of the paradox, and to evaluate the impact of avoidance investment on household health human capital.

Numerous studies have examined the increasing cost of pollution protection to accurately evaluate the socioeconomic losses caused by environmental pollution [1,2]. Related studies have identified that the accumulation of human capital is negatively affected by exposure to environmental pollution in adolescence, thus decreasing later career income, which shows that the harm caused by pollution could last much longer than previously recognized [3]. Moreover, pollution avoidance behavior shows a persistent trend; at least, expenditures on pollution protection are not reduced along with the decrease in pollutants, as previously imagined. The average concentration of PM2.5 in the initial 74 cities was 72 μg/m^3^ in 2013 and 41.8 μg/m^3^ in 2018, presenting a decrease of 42%. However, the average growth rates of the annual sales of anti-smog masks and purifiers in the Chinese market remained above 20% during the same period. This indicates a paradox that has been ignored in previous studies, with few researchers attempting to investigate the issue on the basis of microlevel evidence. More studies are needed to explore pollution protection behaviors in different periods in order to understand why people’s self-protection investment persists after the environment improves and to observe whether the persistent effect is a complement to or substitute for medical expenditure. It is of great significance to accurately evaluate the welfare loss caused by environmental pollution.

According to the theory of health human capital, an individual’s health is produced through the household production function, and the cost includes time, medical care, food, leisure, etc., Production efficiency is affected by factors such as education, environment, and age, which is called the health production efficiency hypothesis [4,5]. The research related to the impact of environmental pollution and self-protection behaviors on an individual’s health is mostly based on health human capital theory and the household production function. The theoretical analysis of self-protection behavior based on the hypothesis of health production efficiency generally argues that highly educated groups produce health or allocate resources to produce health in a more efficient way than less-educated groups, which leads to rational protection choices and positive health outcomes. However, the relationship between self-protection investment and medical expenditure may be substitutional or uncertain. The two expenses may substitute for each other given the constraints of time and disposable income [1,6] while uncertainty arises because self-protection investment is constrained not only by the time cost and material cost but also by the cost of knowledge and information. Furthermore, in addition to health, other welfare aspects of self-protection investment include non-healthy benefits, such as security, dignity and honor [7]. With the development of health human capital studies, researchers have put forward the hypothesis of time preference, which holds that health is not only limited by an individual’s resource endowment but also related to the decision-maker’s risk trade-off between consumption and health, or the discount rate given to future health time [8,9,10].

Based on the theoretical research on the health production model and self-protection behavior, this study aimed not to determine whether smog avoidance investment could bring health benefits to individuals and families but to evaluate the impact of smog avoidance investment on household health care expenditure and personal self-rated health using online survey data from six major cities, Shenyang, Chengdu, Shanghai, Guangzhou, Beijing and Tianjin, and 11 prefecture-level cities in Hebei Province from November 2018 to February 2019. In this paper, the two variables of household health care expenditure and self-rated health are used as measurement indexes, and a treatment effect model is employed to overcome the possible endogeneity bias. The results indicate that smog avoidance investment had no substitution or crowding out effect on household health care expenditure but had a significant positive impact and, over time, it also had a certain persistent effect. Families with higher education levels had higher self-protection investment ratios and health care expenditures. Knowledge of smog hazard and protection investment had a significant interaction effect, which further increased health care expenditures. Moreover, smog avoidance investment significantly improved the probability that individuals assess their health as “good” and “very good”. In addition, subjective risk perception and the health time discount rate index significantly impacted self-rated health. We thus confirm that the smog avoidance investment and health production of Chinese urban residents can be better explained by the time preference hypothesis than by the healthy production efficiency hypothesis. Although previous research based on short-term air quality fluctuation and smog avoidance investment has revealed hidden losses due to air pollution, the social welfare loss caused by air pollution might still be underestimated due to the persistent effect of risk aversion observed in this study.

The main contributions of this paper are as follows: First, we provide an explanation for the persistent effect of smog avoidance investment and further enrich the existing research on pollution protection behavior. To the best of our knowledge, this is the first study to observe the contradiction between the improvement in the air quality along with the increasing pollution protection investment among families in China, and the explanation is given based on microlevel evidence. Second, it uses current data for representative cities after China’s air pollution control efforts achieved significant effects. The health production efficiency hypothesis and the time preference hypothesis are included in the analytical framework to more fully reveal the decision-making behind current urban residents’ pollution protection behaviors. Third, this study provides new practical evidence to facilitate an objective and comprehensive evaluation of the social welfare loss caused by air pollution in order to achieve social consensus and optimize environmental regulation policies.

## 2. Literature Review

Many studies have shown that pollution protection behaviors are widespread. In the short term, individuals adjust outdoor activity time to reduce pollution exposure [11,12,13,14,15]. In the long term, families can avoid the polluted environment by choosing a less-polluted place of residence [16,17,18] or even emigrating abroad [19]. However, for most people, short-term protection is still the first choice. Pollution protection investment behavior, such as wearing anti-smog masks and using air purifiers, can reflect people’s health demands well [20]. Earlier studies on the cost of pollution protection in China mostly infer the hidden loss of air pollution from the relationship between short-term air quality fluctuations and the sales of anti-smog products. According to the data from China’s mainstream e-commerce platform, when air quality reaches the “heavy pollution” or “extremely heavy pollution” level, sales of anti-smog masks and air purifiers can increase by 1.3 to 7.2 times compared to sales during the “good air quality” level [21]. If the number of days of heavy pollution is reduced by 10%, the national savings on mask expenditure alone can be 1.146 billion RMB yuan [2].

Other empirical researchers focus on investigating the factors influencing pollution protection behaviors, considering the health production efficiency hypothesis and time preference hypothesis, because such behaviors are closely related to environmental equity and social welfare policy. However, highly detailed data requirements accompany this kind of research. Hoffmann and Lutz [22] used survey data for the Philippines to characterize health knowledge in various dimensions by constructing a composite index and used generalized propensity score matching to confirm that education level explained 69% of lifestyle differences, which supports the health production efficiency hypothesis. However, they also pointed out that their research did not allow causal inference because the key explanatory variables of education and knowledge may still be endogenous, and their results may show only the importance of knowledge for health as a moderator of education. Bijwaard and Kippersluis [23] used detailed Dutch tracking survey data over a long period to distinguish educational achievement and endogenous ability factors and revealed that the intelligence factor accounted for a substantial portion of health outcomes and educational achievements, such that “smarter people are healthier”. They challenged the interpretation that education itself drives the efficiency of health production. Like educational achievements, risk perception and time preference are endogenous [7,24], and assessment results can be reliable only after the endogeneity is addressed. A Canadian study based on online survey data showed that estimated risk-averting expenditures are 3 times higher when the endogeneity of risk perception is considered than when it is not [25]. Other studies show that the hypothesis of health production efficiency or time preference can only partially explain an individual’s decision-making rules. For example, Wakefield’s research suggesting that vaccines lead to autism is widely distributed in the United Kingdom and the United States, so some parents, especially more highly educated mothers, delay vaccination or refuse to vaccinate their children, which supports the hypothesis of health production efficiency. However, even after the dissemination of information refuting this controversial study, differences in vaccination rates among families at different educational levels still exist, showing a significant educational group persistence effect in the United States, but not in the United Kingdom. This contradictory result may support the time preference hypothesis to some extent [26]. Although many previous studies have sought to evaluate the welfare loss of pollution, few studies have focused on protection behavior and its impact on household medical expenditure. In addition, most papers estimating the hidden costs of pollution defense assume that air pollution is the main reason for the choice of self-protection and that air quality improvement will automatically reduce the cost of self-protection, ignoring the persistent effect or inner mechanism derived from updated information, risk awareness and future health expectations. This leads to the contradiction between the previous self-protection cost evaluation and the updated data of the “anti-smog industry”. The strict atmospheric pollution control policies that began in 2013 in China give us a chance to observe and explore citizens’ pollution protection investment and its impact.

From the perspective of the protective effects, although most studies have confirmed that anti-smog masks and air purifiers have protective effects on the cardiopulmonary system of healthy adults, the conclusions regarding the key indicators for sensitive people with different symptoms are not consistent, and the relevant protective effect studies are based on the strict and correct usage of the products. It is necessary to conduct further dose-response tests and control the influence of confounding factors. Therefore, studies have not yet managed to draw a consistent conclusion [27,28].

Furthermore, previous studies on avoidance behavior present certain limitations, such as missing variables and inadequate endogeneity treatment. Previous studies have mostly used air quality as the endogenous factor and ignored other endogenous factors related to the choice of self-protection behavior. This is due to the availability of data because outdoor activity statistics, social media sign-in data, and e-commerce platform data do not provide information about the detailed individual characteristics and perceptions of users, so such analysis has a high likelihood of missing variables and is challenged to address the endogeneity problem of the decision to engage in self-protection behavior. This article compensates for the above deficiencies.

## 3. Materials and Methods

### 3.1. Treatment Effect Model

The smog avoidance investment behavior discussed in this study basically does not involve time cost, so there is no income constraint and knowledge cost, but the health care investment involves the above three aspects of cost constraints. Therefore, for low-income families, protection investment and medical expenditure may show substitution or crowding out effects, while for high-income families, the two behaviors may present a mutually promoting relationship. From the perspective of risk preference, self-protection investment and medical insurance expenditure may be carried out at the same time to prevent or reduce serious health risks. Of course, it is also possible that even if a household member is in poor health, the household may not purchase anti-smog masks or air purifiers because of economic pressure or simply because of the feeling that their health is not affected by smog and that it is unnecessary to purchase an anti-smog mask or air purifier. In other words, regardless of the situation, household protection investment does not occur randomly but is a self-selection behavior. If OLS regression is used, the results will be biased due to potential unobservable characteristics (such as risk preference) or missing variables, which will affect the impact of investment in smog avoidance. Moreover, smog avoidance investment may affect household medical expenditure and self-assessed health at the same time, so we use a treatment effect model to estimate the impact of smog avoidance investment on medical health expenditure and health outcomes. The treatment effect model proposed by Maddala [29] follows the tradition of Heckman’s sample selection model but differs from it in two respects: first, the dummy variables indicating the processing conditions directly enter the regression equation; second, the regression equation results variables are observed for different selection states. The regression equation is shown as Equation (1):

(1) $$y_{i}=X_{i}^{\prime }\beta +\gamma D_{i}+\varepsilon_{i}$$

where $y_{i}$ is the result variable of individual *i*, $X_{i}\;$is the exogenous covariate, $\varepsilon_{i}$ is the random disturbance term, and $D_{i}$ is the selection result variable of sample *i*. If the smog avoidance investment is carried out, $D_{i}=1,\;$otherwise $D_{i}=0$. It is assumed that the dummy variable $D_{i}\;$ indicating the treat result is determined by the treatment Equation (2):

(2) $$D_{i}=1\left(z_{i}^{\prime }\delta +\mu_{i}\right)$$

where 1(⋅) is an indicative function, and $z_{i}^{\prime }$ variables can overlap with $X_{i}^{\prime }\;$ variables, but instrumental variables, such as $z_{1i}$, are not in $X_{i}^{\prime }$. We further assume that $\mathrm{Cov}\left(z_{1i},\varepsilon_{i}\right)=0$, where $z_{1i}$ affects whether individuals participate in $\;D_{i}$ but does not directly affect the result variable $y_{i}$ (affecting $y_{i}$ only indirectly through $D_{i}$), and the disturbance term $\left(\varepsilon_{i},\mu_{i}\right)\;$ obeys a two-dimensional normal distribution:

(3) $$\left(\begin{array}{c} \varepsilon_{i}\\ \mu_{i} \end{array}\right)\sim N\left[\left(\begin{array}{c} 0\\ 0 \end{array}\right),\left(\begin{array}{cc} \sigma_{\varepsilon }^{2} & \rho \sigma_{\varepsilon }\\ \rho \sigma_{\varepsilon } & 1 \end{array}\right)\right]$$

where ρ is the correlation coefficient of $\left(\varepsilon_{i},\mu_{i}\right)$, the variance of $\mu_{i}$ is normalized to 1 (because $\mu_{i}$ is the disturbance term of the probit model), and ρ≠0, which is the source of the model’s endogeneity. Otherwise, there is no endogeneity, and the consistency can be estimated directly by OLS. For the sample that chooses smog avoidance investment, the conditional expectation of $y_{i}$ is shown in Equation (4):

(4) $$E\left(y_{i}|D_{i}=1,x_{i},z_{i}\right)=x_{i}^{\prime }\beta +\gamma +E\left(\varepsilon_{i}|D_{i}=1,x_{i},z_{i}\right)\;=x_{i}^{\prime }\beta +\gamma +E\left(\varepsilon_{i}|z_{i}^{\prime }\delta +\mu_{i}>0,x_{i},z_{i}\right)=x_{i}^{\prime }\beta +\gamma +\rho \sigma_{\varepsilon }\lambda \left(-z_{i}^{\prime }\delta \right)$$

where λ(⋅) is the inverse Mills function, and the expectation of the result variable of the sample without smog avoidance investment is:

(5) $$E\left(y_{i}|D_{i}=0,x_{i},z_{i}\right)=x_{i}^{\prime }\beta +E\left(\varepsilon_{i}|D_{i}=0,x_{i},z_{i}\right)=x_{i}^{\prime }\beta +E\left(\varepsilon_{i}|z_{i}^{\prime }\delta +\mu_{i}\leq 0,x_{i},z_{i}\right)=x_{i}^{\prime }\beta -\rho \sigma_{\varepsilon }\lambda \left(z_{i}^{\prime }\delta \right).$$

By subtracting (4) and (5), the difference between the conditional expectations of the samples that invest in smog avoidance products and the samples that do not is obtained:

(6) $$E\left(y_{i}|D_{i}=1,x_{i},z_{i}\right)-E\left(y_{i}|D_{i}=0,x_{i},z_{i}\right)=\gamma +\rho \sigma_{\varepsilon }\left[\lambda \left(-z_{i}^{\prime }\delta \right)+\lambda \left(z_{i}^{\prime }\delta \right)\right]$$

Clearly, if we directly compare the average results $y_{i}$ of the two groups, the second term to the right in the above equation will be ignored, resulting in inconsistent estimates (unless *ρ* = 0). To put the treatment group and control group together for regression, the hazard of individual *i* is defined as:

(7) $$\lambda_{i}=\{\begin{array}{ll} \lambda \left(-z_{i}^{\prime }\delta \right) & D_{i}=1\\ \lambda \left(z_{i}^{\prime }\delta \right) & D_{i}=0 \end{array}$$

Equations (4) and (5) are merged into an equation to describe the decision-making of the samples regarding whether to conduct smog avoidance investment:

(8) $$E\left(y_{i}|x_{i},z_{i}\right)=x_{i}^{\prime }\beta +\gamma D_{i}+\rho \sigma_{\varepsilon }\lambda_{i}$$

Equation (8) can be estimated by the two-step method or by the more efficient maximum likelihood estimation (MLE) method performed to obtain all parameters simultaneously. However, since the main equation and the treatment equation involve the same variables, the results using the two-step method may be unstable due to the collinearity problem. In contrast, the MLE method has no collinearity problem, and the estimation result is more reliable. Therefore, this paper employs the MLE method.

In addition, the form of the function for estimating $E\left(y_{i}|x_{i},z_{i}\right)$ needs to be consistent with the characteristics of $y_{i}$*,* and household medical cost is a continuous variable, which can be in the form of a linear function. Self-assessed health is a non-linear function, and we use the ordered probit model that is widely applied in the literature for estimation.

Zhao Zhong and Hou Zhengang’s [30] method for measuring the health demands of urban residents and Sun et al. [31] method for measuring the willingness to pay for air quality are referred to. Moreover, air pollution, pollution warning and weather variables are incorporated into the equation, and the risk of pollution perception, knowledge of smog prevention, discount rate on health (indexed as smoking status and weekly exercise time), age, education level, employment status, household income per capita, and whether the individual has medical insurance are included as control variables. In addition, much evidence shows that social and economic conditions in different cities lead citizens to receive different levels of medical security, which significantly affects their personal health status [32]. Therefore, the per capita GDP of the city, the number of beds in medical institutions per 10,000 people, and regional fixed effects variables are included among the control variables. To address outliers in healthcare expenditure, the censored methods of Bellante and Kogut [33] and Liu Quan [12] are adopted; that is, all 3% outliers are replaced by the closest no-outliers.

### 3.2. Selection of Instrumental Variables

At least one instrumental variable different from the variables in the regression equation should be added to correct the endogeneity bias caused by self-selection in the selection equation of the treatment effect model. From the relevant literature, the instrumental variables method was used to estimate the impact of air pollution on key variables, such as medical insurance demand, medical expenditure, and morbidity. Previous researchers have used rainfall [34], the main wind direction and average wind force [35], the transboundary spillover effects of pollutants [36], the air flow coefficient [37,38] or other variables related to pollutant diffusion or accumulation. However, the treatment effect model is different from the instrumental variable method, and we sought to eliminate the endogeneity of an individual’s non-random selection, so we also referred to the research on selection into the treatment effect, such as the impact of residential mode on happiness [39] and the impact of the channels chosen for agricultural product sales on the sales growth rate [40]. Finally, the local annual average wind speed of the dominant wind direction, the local anti-smog mask adoption rate excluding the sample, and the local ratio of individuals purchasing air purifiers excluding the sample were selected as the instrument variables in the treatment equation. Intuitively, these three variables would not have a direct impact on household medical expenditure or personal self-assessed health but would affect the self-protection decision through possible peer effects or pollutant transmission. Finally, by checking whether the hypothesis of ρ≠0 was rejected, we could determine whether the selected structural equation setting was reasonable.

### 3.3. Sampling and Data Survey

**Figure 1:**
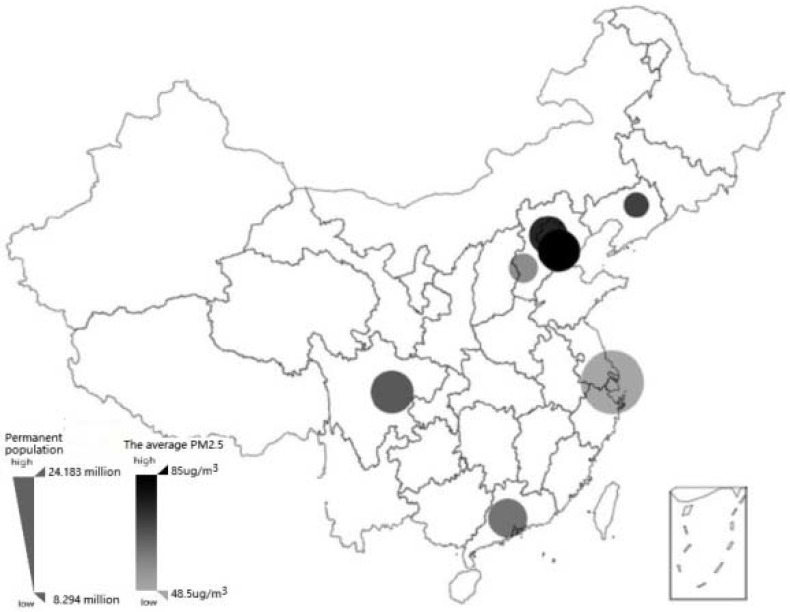
Sample distribution and the relative situation of the local permanent population and pollution in 2017. It shows the distribution of 7 cities at the provincial capital level and above among the sample cities in China. The circle area represents the relative proportion of the permanent population in the region, and the shading represents the relative value of the local average PM2.5 concentration in 2017.

Due to the budget constrain, we sampled 17 representative cities from different development regions in China to conduct survey data collection. The selected cities are representative of China’s geographical and economic development in the Northeast (Shenyang), Beijing-Tianjin-Hebei (Beijing, Tianjin, Shijiazhuang, Tangshan, Qinhuangdao, Handan, Xingtai, Baoding, Zhangjiakou, Chengde, Cangzhou, Langfang, Hengshui), Western (Chengdu), Pearl River Delta (Guangzhou) and Yangtze River Delta (Shanghai) regions in order to increase the representativeness of the sample and the statistical power of the research results. All of the 17 cities are in the prevention and control areas listed by the “Ten rules of Qi”; 10 cities were key prevention control areas, and 7 cities were general control zones. (Table 1 for specific divisions). In each city, a target sample of the population 18 years old and over was determined according to the proportion of permanent residents. Sample distribution and the relative situation of the local permanent population and pollution in 2017 are displayed in Figure 1.

**Figure 2:**
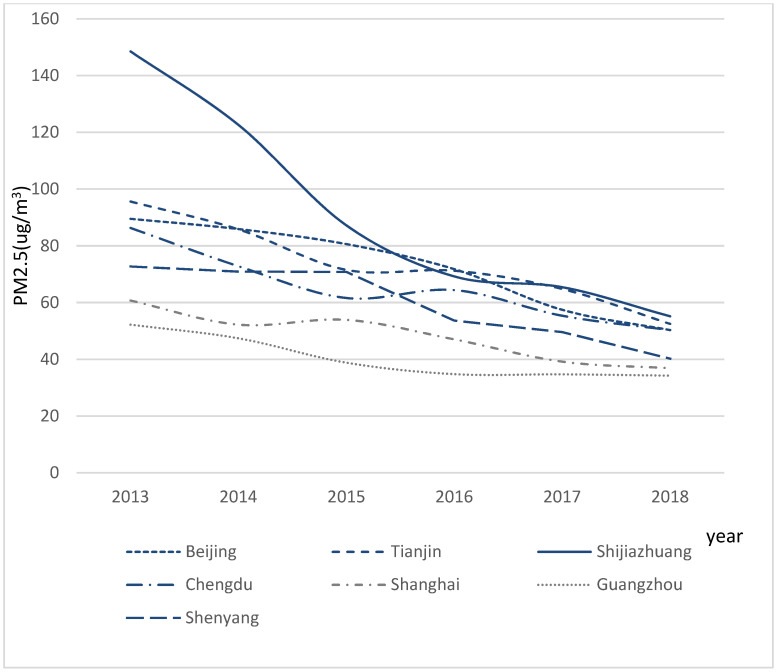
The average annual PM2.5 of 3 municipalities directly under the Central Government and four provincial capitals from 2013 to 2018. Five of these seven cities are the top 10 cities in China, and they are distributed in the major economic development areas, which are representative of the changes of urban air quality in China. This shows the change in the annual mean value of PM2.5 in seven cities during 2013–2018 after the “Ten Rules of Qi” was implemented. At the beginning of 2013, the annual average PM2.5 of all cities exceeded the national standard of 36 mg/m^3^, and the degree of pollution in each city varied substantially. For instance, in 2013, the average PM2.5 concentration in Shijiazhuang reached 148.5 mg/m^3^, nearly three times the level in Guangzhou, 52.2 mg/m^3^, which presented the best air quality in the same period. However, by 2018, the air quality of every city had improved significantly and showed obvious convergence characteristics.

Figure 2 not only indicated that the environmental governance was effective but also might be consistent with the internal structural changes associated with economic development [41].

From November 2018 to February 2019, we conducted a questionnaire survey through the online survey platform “Questionnaire Star”. The platform sent out invitations to participate in the survey in the form of website information and WeChat connections until the predetermined sample size in the local area was reached. The Questionnaire Star platform (www.wjx.cn accessed on 13 January 2018) is the largest company specialized in network surveys in China, with 2.6 million sample database members in November 2018. The website sends questionnaire links including various business surveys to registered users who meet the sample requirements. Registered users can choose their own answers. In return, the respondents will receive credits or equivalent monetary lottery tickets, which can be exchanged for prizes or telephone charges.

To ensure the reliability of the online questionnaire data, we took the following control measures: first, the platform randomly sampled users according to the users’ attribute information, invited them to answer the questionnaire, ensured the authenticity of the source of the sample location through the IP address, computer ID and user name information, and prevented repeat responses. Second, invalid questionnaires were eliminated through rule control, such as setting option permissions, including trap test questions and determining a minimum answer time. The questionnaires that were completed in less than 8 min and that failed the consistency checks were eliminated to improve the authenticity of the data. A total of 2618 questionnaires were collected during the survey period, and 2540 valid questionnaires were obtained after the invalid questionnaires were removed. Finally, to ensure the representativeness of the sample data, we referred to Li Yichong et al. [42] and the STATA weight design processing method5 and adjusted the sample weight according to the number of permanent residents and the age and gender distribution of Internet users in “The 41st Statistical Report on Internet Development in China”. The following statistics and regression measurements were added with weight factors. Table 1 shows the regional distribution of the samples.

We believe that the sample has acceptable reliability for the following reasons: first, if Internet users spend 10–30 min answering a questionnaire without coercion, then their responses are likely to have been given carefully. Some questionnaires could thus be eliminated considering the time the respondents took to complete them. Second, the sample users could obtain bonus points by answering questions, which could improve the sample participation rate and the balance of the sample distribution to a certain extent. Third, the respondents did not face the interviewees directly. By contrast with face-to-face interviews, participants who do not face psychological influences such as vigilance, social expectation and political correctness may make it easier for them to express their ideas frankly in online surveys.

### 3.4. Variable Description and Analysis

The questions in the online questionnaire mainly cover personal and household demographic information, socioeconomic status, health status and medical expenditure, environmental perception, etc., Table 2 presents the description of relevant variables.

Table 3 shows the basic statistics of all variables in the econometric model.

As shown in Table 3, the mean age of the sample was 31.23 years old, of which the proportion of women (58.3%) is higher than that of men (41.7%), 63% of the sample were married, the mean household population was 3.99, and the education level was above high school or college. The per capita income of the average household was 28,300 RMB yuan in 2017, and the proportion of the sample with a chronic disease diagnosed by a doctor was approximately 10%. The mean value of the natural logarithm of annual household medical expenditure (medcost) was 7.17 (1012.32 RMB yuan) in 2017, which was lower than the average level of national urban residents’ medical and health expenditure of 1777.4 RMB yuan in 2017. The mean value of self-rated health (health) was 3.584, which was between “average” and “relatively healthy”. The mean household expenditure on anti-smog masks or air purifiers (Exp) was 0.806. In fact, 73.58% of households used anti-smog masks, and 35.59% used air purifiers. This showed that quite a few families had made smog avoidance investments to address air pollution. From the perspective of air pollution, the average number of severely polluted days of grade 5 in the 17 cities exceeded two weeks in 2016 and 2017, but the time of serious pollution decreased. Shijiazhuang and Hengshui reached 46 days in 2016, while the most polluted cities in 2017 were Handan and Baoding, with 32 days. The cumulative number of days of severe pollution still exceeded one month, indicating that the air pollution in some areas remained quite serious.

For the subjective perceived smog severity (fogbad), the mean value was 2.370, which is between “serious” and “average” but indicates that the respondents were inclined to think that the pollution was serious. This reflects that the sampled residents were still dissatisfied with the air quality and had a high-risk awareness. From the perspective of health habit variables, the overall health consciousness of the sampled citizens in the surveyed cities was relatively high, and the average weekly exercise time was more than 4 h. On the other hand, for the group that did not invest in smog avoidance, there were significant differences in terms of individual and household endowment indicators, risk perception, smog prevention knowledge and time preference variables. The differences for all variables except for the average age, household size, number of elderly and children in the household, chronic diseases and local medical public resources were significant. The sample group that had not made smog avoidance investments considered the local pollution status to be less serious and had lower health awareness, a higher smoking ratio and less exercise time per week. In terms of indicators that reflect the per capita household income, medical insurance coverage ratio, employment status and local economic conditions, the samples that did not invest in smog avoidance also presented significantly lower values than the investment sample group, with 39% lower medical expenditure, on average, and lower self-rated health. These results demonstrate that the decision to invest in smog avoidance is not random, and it is reasonable to use the treatment effect model for estimation.

## 4. Results

### 4.1. Estimation Results of the Treatment Effect Model

Equation (8) is estimated to analyze the impact of air pollution and smog avoidance investment on medical expenditure. The results are shown in Table 4. Column (1) is the basic model, and columns (2), (3) and (4) add the smog awareness, smog subjective risk perception and health time discount rate variables, respectively, to the basic model of column (1). The results show that smog avoidance investment always has a positive impact on household health care investment and remains one of the largest influencing factors even though the extent of the impact decreases when additional control variables are included. Although protective equipment may be unable to effectively reduce the harm due to air pollution depending on the frequency of use, correctness of the usage method and individual heterogeneity, the probability that the use of protective equipment increases medical expenditure is low, which is difficult to explain from the perspective of the health production efficiency hypothesis. However, according to the time preference hypothesis, an individual’s self-protection investment is based on the expectation of future health utility and the protection against the risk of severe damage. Therefore, smog avoidance investment and health care expenditure are not substituted, and there is no crowding out effect of smog avoidance investment on health care expenditure.

The coefficients of the pollution index are also in line with the time preference hypothesis, and the mean PM2.5 concentrations in 2016 and 2017 have no significant impact on medical expenditure. However, the smog awareness variables have a significant impact on health care expenditure, but the impact direction varies across periods, with a significant positive impact in 2016 and a negative impact in 2017. A possible explanation is that smog avoidance behavior and investment have cumulative effects over time. The smog awareness variable for 2016 indicates that the sample considered pollution to be severe and this induces an increase in medical expenditure but not in smog avoidance investment. With the diffusion of smog hazard information or related knowledge, the protective effect appears, and the medical cost is reduced. The subjective smog risk perception variables have a significant impact on medical expenditure if the household perceives the local smog not to be severe and its health care expenditure is low. The better knowledge of smog avoidance a household has, the higher the household health care expenditure. Moreover, if the two variables are ignored, the estimated impact of smog avoidance investment greatly increases. The coefficients of the health discount rate reflected by regular exercise show that the discount rate has a significant positive impact on medical expenditure, which is consistent with the time preference hypothesis. The sample with a higher education level and a higher household income level spent more on medical care, which is consistent with the empirical findings of most health production functions.

The correlation coefficients of the residual terms in the two equations are between −0.5881 and −0.3982 in models (1)–(4), and the null hypothesis that the correlation coefficient is 0 is rejected. ρ<0 shows that the unobservable factors in the model reduce the probability of smog avoidance investment but increase household health care expenditure. The impact of smog avoidance investment on health care expenditure would be underestimated by OLS estimation, so it is suitable to use the treatment effect model.

### 4.2. Heterogeneity Analysis of the Impact of Smog Avoidance Investment on Household Health Care Expenditure

**Figure 3:**
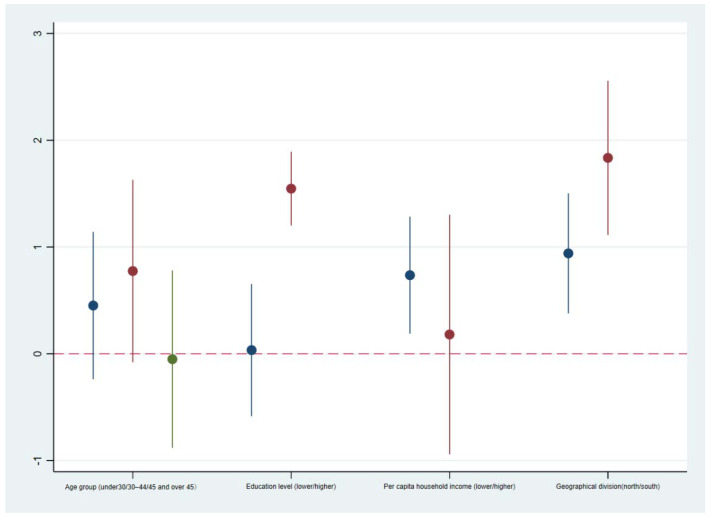
Heterogeneity effect of smog avoidance investment on household health care expenditure. The Exp parameter (±1.96 SE) estimated for each group of samples in Table 4 model (4) is shown.

The paper divides the samples in different subgroups by age, education level, household income, and geographic region to conduct a heterogeneity analysis of the impact of smog avoidance investment on household health care expenditure. Referring to previous methods for grouping samples according to age [11], education [36], household income [38] and geographical area and considering the actual distribution of the samples, we divided the samples into three groups by age: under 30 years old, 30–44 years old and 45 years old and older. From 1989 to 2003 (such that undergraduates who first enrolled in 2000 had completed their college education), the group having below senior high school education and the group having senior high school education or above were defined as the low degree and high degree groups, respectively. From 2003 to 2011, the group with below university-level education and the group with university-level education or above were defined as the low education and high education groups, respectively. The sample was divided into a relatively low-income group and a relatively high-income group according to the median per capita household income. The sample was further divided into a northern group and a southern group according to the Huai River boundary. The four groups of samples are regressed according to model (4) in Table 4. Figure 3 shows the estimated Exp parameter (±1.96 SE) for each group of samples. The results show that smog avoidance investment has a heterogeneous impact on household medical expenditure across groups. Although most of the coefficients are positive, the impacts for the middle-aged and elderly group (45 years old and above), lower-educated group and high-income group are small and not significant, which can be explained by their risk perception of air pollution, health information utilization, living conditions and other factors. It also further illustrates that air pollution protection strategies vary across individuals. This further reminds us that people’s smog avoidance investment is based on health expectations and not just current utility.

### 4.3. Mechanism Analysis

#### 4.3.1. Smog Avoidance Investment and Perceived Health

To further verify that pollution protection investment may be based on an individual’s perception and future health time utility, we used self-rated health, commonly used in the literature, as a dependent variable and investigated the impact of smog avoidance investment on it. Table 5 shows the estimated results. The reg column presents the results of OLS regression with self-rated health as a continuous variable. Oprobit is the results of the ordered probit regression, and the third column includes Exp as the endogenous processing variable, which is the regression result of the ordered probit processing effect.

The estimators of the three models in Table 5 indicate that smog avoidance investment has a significant positive impact on self-rated health. The risk perception variables, health discount rate indicators and chronic diseases also have a significant impact, and the impact direction is consistent with the impact on health care expenditure, further indicating that the self-protection investment decision of Chinese citizens is based on risk perception and health expectations.

Because the meaning of the parameters of the treat oprobit model is not intuitive, the results in Table 5 can only give limited information in terms of significance and parameter symbols, and the coefficients are not comparable. Therefore, we calculate the discrete marginal effect and continuous marginal effect of smog avoidance investment on self-rated health following the method of Lian Yujun et al. [39]. The results are shown in Table 6.

For the endogenous explanatory variable Exp, the discrete marginal effect calculates the probability of the explanatory variable in each value when Exp = 1 or Exp = 0 while keeping other variables at the mean value. The difference between the two probabilities is the discrete marginal effect of Exp; in other words, when other conditions are at the average level, only the state of smog avoidance investment decision changes, and the discrete marginal effect indicates the probability that each value of the explained variable changes. The continuous marginal effect of Exp indicates the various values the probability of self-assessment of health takes when the probability of Exp = 1 changes.

The discrete and continuous marginal effects of the smog avoidance investment decision on self-rated health are similar (Table 6). In terms of the discrete marginal effect, when other variables are at the mean value, the probability of self-rated health being “very poor” and “poor” is reduced by 9.17% and 19.99%, respectively; when the state of smog avoidance investment changes from No to Yes, the probability of “good” and “very good” self-rated health increases by 26.17% and 16.31%, respectively. Considering the descriptive statistics in Table 3, the prediction probability of the model is close to the real distribution. The mean value of health is 3.584, which is between “average” and “good”. The mean value for the sample not engaging in self-protection investment is lower than 3.5, the mean value for the group making self-protection investment is higher than 3.5, and the difference is 0.171. Compared with the former group, the group making self-protection investment has a higher probability of indicating “good” and “very good” self-rated health.

Although the conceptual explanations for the continuous marginal effect and the discrete marginal effect differ, the two show similar results. When all variables are at the mean value and the probability of self-protection investment Prob(exp=1|x) increases Δ, the probability of “very poor” self-assessed health Prob(health=1|x) decreases by 0.0686Δ, and the probability of “very good” self-assessed health Prob(health=5|x) increases by 0.2478Δ. The values in Table 6 indicate that for a household with other conditions at the average level, smog avoidance investment will dramatically increase the probability that respondents indicate “good” and “very good” health status, reduce the probability of “average” health conditions, and to a lower extent reduce the probability of “poor” and “very poor” health conditions.

#### 4.3.2. Analysis of the Interaction Effect of Smog Avoidance Investment, Information Application Ability and Household Income Level

Both the healthy production efficiency hypothesis and the time preference hypothesis take an individual’s information utilization ability or the knowledge information input as important factors. After more than five years of smog harm, related health knowledge dissemination and consumption behavior transmission, people’s self-protection behavior and the cumulative effects of their behaviors may be very different than they were at the beginning of the smog outbreak in 2012–2014.

To examine whether the interaction between related factors and pollution protection behavior impacts the dependent variables, the interaction effects of the smog avoidance investment decision with information utilization ability and household self-assessed social variables are investigated. The results are shown in Table 7; column (1) and column (2) present the results when Exp ∗ Knowledge and Exp ∗ position are added to the estimation equation. The results demonstrate that both interactions significantly increase medical and health expenditures. Compared with the effect in Table 4, the main effect of self-protection investment is reduced when the level of protection knowledge is zero and the self-rated household income level is the lowest.

**Figure 4:**
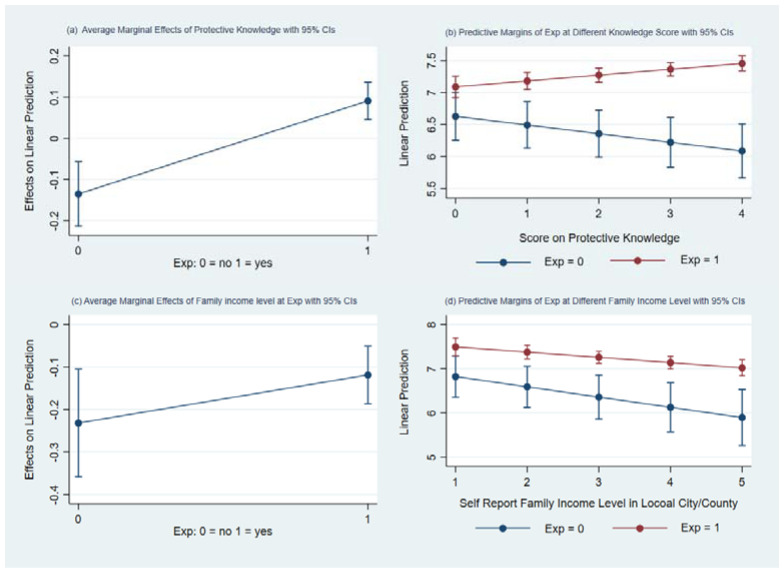
Interaction effect of smog avoidance, information application ability and self-rated social stratification.

Furthermore, Figure 4 shows the marginal effects of the interaction between smog avoidance investment and the information utilization ability variable or self-rated household income level. Figure 4a shows that with other conditions held constant and by comparison with families without self-protection investment, families with average information utilization ability significantly increase their health expenditure when they invested in smog avoidance. Figure 4b shows that when families make self-protection investment, their health expenditures can further increase because of their information utilization ability, and when families do not invest, their medical and health expenditures can be reduced, which shows that information utilization abilities have effects in different directions depending on the situation. However, the interaction between self-rated household income level and self-protection investment is different. Compared with households without investment, if families making smog avoidance investment rate the local pollution at an average level, they may increase their medical and health expenditures to strengthen their protection from pollution, but as the income level increases, both families with and without self-protection investment present lower household medical and health expenditures to varying degrees. This may indicate that high-income groups have other alternatives for protecting themselves from pollution, such as work environment or residence choice and outdoor exposure time.

## 5. Conclusions and Policy Implications

Since the implementation of strict environmental regulations in China from 2013, a paradox has emerged in households’ health capital investment. The households’ investment in pollution avoidance has been increasing while the air quality has been improving remarkably. This paper provides an explanation to this paradox. Based on online survey data of six major cities and 11 cities at the prefecture level and above in Hebei Province from November 2018 to February 2019, we investigated the relationship between air pollution avoidance investment, household health care expenditure and self-rated health. The results suggest that smog avoidance investment has no crowding out effect on household health care expenditure but has a significant positive effect that varies across ages, income levels and education levels. Perceived pollution intensity, pollution prevention knowledge and future health time preference have significant promoting effects on investment decision-making and health care expenditure. Moreover, air pollution protection investment enhances the probability that urban residents indicate “good” and “very good” self-assessed health by 26.17% and 16.31% respectively, and the subjective risk perception and the discount rate of health time significantly improve self-assessment health. Furthermore, there are significant interaction effects between smog hazard knowledge, household income level and self-protection investment, which increase health care expenditure additionally, but with the increase in income level, the promotion effect is gradually reduced but still significant. Compared with the hypothesis of health production efficiency, the time preference hypothesis can better explain the smog avoidance investment and health demands of Chinese urban residents. After more than five years of smog hazard information dissemination and consumer behavior transmission, people’s choice of self-protection behavior has come to reflect risk aversion and health expectations for the future. Short-term and medium-term air quality improvement cannot reverse the continuous pollution protection costs.

The research results remind us that although previous research on short-term air quality fluctuation and smog avoidance investment reveals the hidden costs of air pollution, the social welfare loss caused by air pollution may still be underestimated. Once the environment is destroyed, not only will the process of restoring “blue water and blue sky” take a long time, but the changed behavior brought by people’s heightened risk awareness will also cause a persistent economic burden and welfare reduction. The results provide a new evidence reference for the cost–benefit evaluation for policy-making. Considering the underestimation of the hidden cost of air pollution, control policy implementation should be stricter and more sustainable. On the other hand, it also indicates that with the development of the social economy, people’s willingness to pay for a clean environment and good health is becoming stronger, which is helpful for achieving social consensus and continuously strengthening and optimizing environmental regulation policies to improve the ecological environment. Furthermore, air pollution induced different health outcomes due to the different avoidance investments, thus creating new environmental inequalities. It is necessary to formulate environmental behavior encouragement and environmental cost-sharing policies in the future.

Although the impact of unobservable factors is reduced by treatment effect models, this study is still essentially observational and has some drawbacks. In different periods, people’s smog avoidance investment may vary in intensity according to their migration status and health status, which would impact household medical expenses. Moreover, the interaction between some control variables is complex and difficult to observe. In the future, the difference in difference (DID) or regression discontinuity (RD) would be used based on the multi-period survey data to reduce the possible influence of confounding factors and missing variables in the aspect of the casual inference method. In terms of research content, we will carry out research on health human capital, avoidance behavior and environmental inequality to provide an evidence reference for prevention and control of avoidance behavior other than pollution from the perspective of health benefits. From the perspective of maintaining social equity, we will provide a theoretical basis and evidence reference for enriching the choice of poverty relief tools.

## Figures and Tables

**Table 1:** Sample distribution.

Region	City	Prevention and Control Level	Permanent Resident Pop in 2017 (Ten Thousand People)	Number of Samples Collected (Persons)	Number of Valid Samples (Persons)	Valid Sample Ratio (%)
Beijing-Tianjin-Hebei	Beijing	Important	2170.70	359	342	95.3
	Tianjin	Important	1556.87	226	216	95.6
	Shijiazhuang	Important	1087.99	164	159	97.0
	Tangshan	Important	789.70	116	113	97.4
	Qinhuangdao	Common	311.08	47	46	97.9
	Handan	Common	951.11	141	139	98.6
	Xingtai	Common	735.16	105	103	98.1
	Baoding	Important	1046.92	150	145	96.7
	Zhangjiakou	Common	443.30	61	60	98.4
	Chengde	Common	356.50	41	41	100.0
	Cangzhou	Common	755.49	101	99	98.0
	Langfang	Important	474.10	73	70	95.9
	Hengshui	Common	446.04	68	65	95.6
Yangtze River Delta	Shanghai	Important	2418.33	403	396	98.3
Pearl River Delta	Guangzhou	Important	1449.84	226	221	97.8
Western	Chengdu	Important	1604.50	231	222	96.1
Northeast	Shenyang	Important	829.40	106	103	97.2
Total	Total		17,427.03	2618	2540	97.0

Notes: The prevention and control levels are based on the “Ten Rules of Qi” and are divided into important and common. Other data are calculated according to the sample survey data collected by the research group.

**Table 2:** Description of variables.

Variable Type	Variable Name	Description
Explained variable	Annual medical expenditure of household (medcost)	Unit: yuan, natural logarithm
	Health self-rated (health)	How do you feel about your health compared to your peers? 5 = very healthy; 4 = relatively healthy; 3 = average; 2 = relatively poor; 1 = very poor
Endogenous explanatory variable	Protection investment, whether purchased protective equipment or not (Exp)	1 = yes; 0 = no
Instrumental variables	Mean value of local mask protection (mask_m)	
	Mean value of local air purifier protection (aircleaner_m)	
	Average wind speed of the local dominant wind direction (wind)	Unit: m/s, take natural logarithm
Exogenous explanatory variables	Size of household (hodsize)	unit: PCS (Pieces)
	gender (gender)	1 = male; 0 = female
	Age (age)	Unit: years
	Educational attainment (edu)	Unit: 1 = primary school, 2 = junior high school, 3 = senior high school, 4 = junior college, 5 = undergraduate and above
	Marriage status (married)	With spouse = 1; without spouse = 0
	Annual per capita income (income)	Unit: yuan, natural logarithm
	Whether the individual has medical insurance (insur)	1 = yes; 0 = no
	Number of people over 60 in the household (elder)	unit: PCS
	Number of people under 18 in the household (kid)	unit: PCS
	Whether employed (job)	1 = yes; 0 = no
	Smoking status (smoking)	1 = yes; 2 = no
	Average exercise time per week (excise)	Hours/week
	Whether has a chronic disease diagnosed by a doctor (disease)	1 = yes; 0 = no
	Whether feels the severity of local smog (fogbad)	Considered severity of local smog: 1–5, 1 = very serious, 5 = not serious at all
	Smog avoidance knowledge (knowl)	0–5 points
	Relative position in terms of household income at the local level (position)	1–5, 1 = far below average, 5 = far above average
	GDP per capita of the local city in 2017 (GDPPC)	Unit: yuan, take natural logarithm
	Number of hospital beds per 10,000 people in the city or district in 2017	Unit: piece
	Average PM2.5 concentration in a year in the local city (PM2.5)	Unit: mg/m^3^
	Number of severe pollution warnings in a year in the city (L5AQI, Air Quality Index)	Unit: time
	Local annual precipitation days (precip)	Unit: day
	Local annual cloudy days (cloudy)	Unit: day

**Table 3:** Descriptive statistical analysis of variable.

Variable	Whole Sample (*n* = 2540)				Exp = 0 (*n* = 494)		Exp = 1 (*n* = 2046)		Mean Difference
	Mean Value	Standard Deviation	Minimum	Maximum	Mean Value	Standard Deviation	Mean Value	Standard Deviation	
medcost	7.170	1.130	5.120	9.100	6.860	1.120	7.250	1.120	−0.39 ***
health	3.584	0.864	1	5	3.447	0.880	3.617	0.857	−0.169 ***
Exp	0.810	0.400	0	1					
PM2.5_16	50.95	13.06	22	73	48.61	14.25	51.51	12.69	−2.91 ***
PM2.5_17	45.96	12.40	25.80	69	44.71	13.38	46.27	12.14	−1.56 **
L5AQI_16	18.62	14.10	0	46	16.45	14.79	19.14	13.88	−2.69 ***
L5AQI_17	14.09	10.40	0	32	12.95	11.12	14.37	10.20	−1.41 ***
fogbad	2.370	1.080	1	5	2.590	1.110	2.320	1.070	0.27 ***
smoking	1.810	0.390	1	2	1.760	0.430	1.820	0.380	−0.06 ***
excise	4.810	7.760	0	70	3.770	6.590	5.070	7.990	−1.29 ***
edu	4.370	0.920	1	5	4.080	1.090	4.440	0.870	−0.36 ***
gender	0.420	0.490	0	1	0.490	0.500	0.400	0.490	0.09 ***
married	0.630	0.480	0	1	0.560	0.500	0.640	0.480	−0.09 ***
age	31.23	9.280	18	75	30.97	10.10	31.30	9.070	−0.330
famsize	3.990	1.210	1	7	4.010	1.220	3.980	1.210	0.0300
income	10.25	0.990	8.010	12.02	9.900	0.950	10.34	0.980	−0.44 ***
elder	0.900	0.960	0	5	0.940	1	0.890	0.950	0.0500
kids	0.640	0.720	0	3	0.600	0.770	0.650	0.700	−0.0500
position	2.830	0.750	1	5	2.590	0.740	2.890	0.730	−0.30 ***
insur	0.770	0.420	0	1	0.690	0.460	0.790	0.410	−0.10 ***
party	0.200	0.400	0	1	0.140	0.350	0.220	0.410	−0.07 ***
job	0.770	0.420	0	1	0.670	0.470	0.790	0.410	−0.12 ***
disease	0.100	0.290	0	1	0.0900	0.290	0.100	0.300	−0.0100
GDP	1.950	0.700	0.880	3.470	1.890	0.690	1.960	0.710	−0.07 *
Mbed	4.060	0.450	3.070	5.620	4.090	0.430	4.050	0.450	0.0400
mintem_16	11.21	4.470	3.080	21.65	11.91	5.070	11.04	4.290	0.87 ***
precip_16	115.9	52.67	56	229	123.5	54.42	114.0	52.08	9.54 ***
cloudy_16	49.90	23.27	2	103	46.43	23.46	50.74	23.15	−4.31 ***
mintem_17	10.771	4.118	3.179	20.39	11.42	4.809	10.62	3.919	0.797 ***
precip_17	96.84	46.91	45	197	105.2	50.57	94.84	45.78	10.32 ***
cloudy_17	41.77	23.88	2	106	37.57	23.79	42.78	23.80	−5.21 ***

Notes: *, ** and *** denote 10%, 5% and 1% significant levels, respectively. The GDP per capita in 2017 is calculated according to the statistical yearbook, the number of days above AQI level 5 and the average value of PM2.5 concentration in 2017 are calculated by the public data of the Ministry of Ecology and Environment of the People’s Republic of China, and other data are collected by the online survey of the research group. PM2.5_16 represents average PM2.5 in 2016. PM2.5_17 represents average PM2.5 in 2017. L5AQI_16 represents average L5 AQI in 2016. L5AQI_17 represents average L5 AQI in 2017.

**Table 4:** Impact of smog avoidance expenditure on household health care expenditure.

Variable	Annual Per Capita Health Care Expenditure of Household			
	(1)	(2)	(3)	(4)
Exp	0.8983 ***	1.0513 **	0.9171 ***	0.7401 **
	(0.2855)	(0.4410)	(0.3165)	(0.3717)
PM2.5_16	0.0147	−0.0048	−0.0003	−0.0030
	(0.0110)	(0.0122)	(0.0182)	(0.0178)
PM2.5_17	−0.0137	−0.0098	−0.0105	−0.0081
	(0.0172)	(0.0143)	(0.0191)	(0.0186)
L5AQI_16		0.0403 ***	0.0371 *	0.0381 *
		(0.0145)	(0.0212)	(0.0207)
L5AQI_17		−0.0458 ***	−0.0470 **	−0.0478 **
		(0.0109)	(0.0190)	(0.0186)
fogbad	−0.0521 **		−0.0423 *	−0.0519 **
	(0.0229)		(0.0230)	(0.0225)
knowledge	0.0408 *		0.0473 **	0.0447 **
	(0.0214)		(0.0219)	(0.0220)
smoking	0.0953 *	0.0971		0.0969 *
	(0.0571)	(0.0758)		(0.0561)
excise	0.0136 ***	0.0132 ***		0.0139 ***
	(0.0027)	(0.0036)		(0.0027)
edu	0.1463 ***	0.1607 ***	0.1395 ***	0.1547 ***
	(0.0281)	(0.0328)	(0.0281)	(0.0282)
gender	0.0351	0.0362	0.0812	0.0211
	(0.0523)	(0.0607)	(0.0504)	(0.0545)
married	0.1210 *	0.1171	0.0940	0.1194 *
	(0.0697)	(0.0994)	(0.0700)	(0.0685)
age	0.0027	0.0032	0.0033	0.0027
	(0.0024)	(0.0039)	(0.0025)	(0.0024)
famsize	−0.1215 ***	−0.1231 ***	−0.1223 ***	−0.1180 ***
	(0.0224)	(0.0267)	(0.0226)	(0.0224)
income	0.2370 ***	0.2314 ***	0.2335 ***	0.2429 ***
	(0.0299)	(0.0360)	(0.0306)	(0.0312)
elder	0.0654 ***	0.0633 *	0.0686 ***	0.0652 ***
	(0.0252)	(0.0328)	(0.0253)	(0.0247)
kid	0.0930 **	0.1021 **	0.0867 **	0.0959 **
	(0.0401)	(0.0509)	(0.0403)	(0.0394)
position	−0.1386 ***	−0.1515 ***	−0.1253 ***	−0.1368 ***
	(0.0326)	(0.0584)	(0.0331)	(0.0328)
insur	0.4289 ***	0.4261 ***	0.4426 ***	0.4318 ***
	(0.0520)	(0.0686)	(0.0527)	(0.0520)
party	0.0401	0.0225	0.0409	0.0410
	(0.0585)	(0.0580)	(0.0589)	(0.0579)
job	0.0855	0.0759	0.0941	0.1033 *
	(0.0578)	(0.0790)	(0.0594)	(0.0613)
disease	0.3334 ***	0.3502 ***	0.3171 ***	0.3426 ***
	(0.0724)	(0.0736)	(0.0727)	(0.0715)
GDP	−0.0300	−0.0719	−0.0715	−0.0688
	(0.0627)	(0.0571)	(0.0659)	(0.0644)
Mbed	−0.1492 *	−0.1306 **	−0.1388 *	−0.1295
	(0.0765)	(0.0631)	(0.0831)	(0.0812)
i.city	√	√	√	√
weth	√	√	√	√
cons	√	√	√	√
/				
athrho	−0.4966 ***	−0.5881 **	−0.4968 **	−0.3982 *
	(0.1803)	(0.2752)	(0.1989)	(0.2300)
lnsigma	0.0398	0.0585	0.0464	0.0218
	(0.0351)	(0.0553)	(0.0382)	(0.0378)
N	2540	2540	2540	2540
athrho				
chi2_c	7.5860	4.5655	6.2404	2.9986

Notes: *, **, and *** denote 10%, 5% and 1% significance levels, respectively, and robust standard errors in parentheses.

**Table 5:** Impact of smog avoidance investment on self-rated health.

Variable	Self-Rated Health		
	Reg	Oprobit	Treat Oprobit
exp	0.1097 **	0.1457 **	1.1298 ***
	(0.0454)	(0.0584)	(0.2008)
PM2.52016	−0.0108	−0.0136	−0.0173
	(0.0134)	(0.0170)	(0.0222)
PM2.52017	−0.0086	−0.0128	−0.0124
	(0.0153)	(0.0198)	(0.0248)
L5AQI2016	0.0423 ***	0.0539 ***	0.0504 **
	(0.0158)	(0.0196)	(0.0247)
L5AQI2017	0.0052	0.0062	0.0103
	(0.0148)	(0.0188)	(0.0204)
fogbad	0.0500 *	0.0660 *	0.0631 **
	(0.0278)	(0.0376)	(0.0308)
knowledge	−0.0182	−0.0225	−0.0468 *
	(0.0255)	(0.0339)	(0.0265)
smoking	0.0059	0.0071	0.0116
	(0.0521)	(0.0697)	(0.0775)
excise	0.0157 ***	0.0219 ***	0.0183 ***
	(0.0029)	(0.0044)	(0.0043)
EDU	0.0527	0.0647	0.0375
	(0.0319)	(0.0411)	(0.0358)
other covariates	√	√	√
con	√	√	√
atanhrho_12			−0.6662 ***
			(0.1627)
Rho_12			−0.5825 ***
			(0.1075)

Notes: *, **, and *** denote 10%, 5% and 1% significance levels, respectively, and robust standard errors in parentheses.

**Table 6:** The discrete and continuous marginal effects of the smog avoidance investment decision on self-rated health.

Variable	Discrete Marginal Effect			Continuous Marginal Effect
	Exp = 0	Exp = 1	Diff	
Self-rated health				
Very poor	0.0996	0.0079	−0.0917	−0.0686
Poor	0.2603	0.0604	−0.1999	−0.1417
Average	0.3949	0.2616	−0.1333	−0.1696
Good	0.224	0.4857	0.2617	0.1322
Very good	0.0213	0.1844	0.1631	0.2478

**Table 7:** Interaction effect of smog avoidance with information application ability and self-rated income level.

Variable	Average Household Health Care Expenditure	
	Protection Knowledge (1)	Self-Rated Level (2)
exp	0.4608 *	0.5592 *
	(0.2520)	(0.3328)
Exp#knowledge	0.2261 ***	
	(0.0435)	
Exp#position		0.1130 *
		(0.0672)
knowledge	−0.1351 ***	0.0400 *
	(0.0399)	(0.0216)
position	−0.1470 ***	−0.2315 ***
	(0.0323)	(0.0646)
other covariates	√	√
cons	√	√
	(1.2975)	(1.2958)
athrho	−0.5344 ***	−0.4740 **
	(0.1526)	(0.1970)
athrho chi2_c F Hansen J statistic(*p*=)	12.2693 16.01 0.5844	5.7927 9.38 0.9563

Notes: *, **, and *** denote 10%, 5% and 1% significance levels, respectively, and robust standard errors in parentheses.

## Data Availability

The data used in this study are available on reasonable request from the corresponding author.
